# Associations between media parenting practices and early adolescent consumption of R-rated movies and mature-rated video games

**DOI:** 10.1186/s12887-024-05367-w

**Published:** 2025-02-04

**Authors:** Jason M. Nagata, Karen Li, Shirley S. Sui, Jonanne Talebloo, Christopher D. Otmar, Iris Yuefan Shao, Orsolya Kiss, Kyle T. Ganson, Alexander Testa, Jinbo He, Fiona C. Baker

**Affiliations:** 1 Department of Pediatrics, University of California, San Francisco, 550 16th Street, 4th Floor, Box 0503, San Francisco, CA 94143 USA; 2 Center for Health Sciences, SRI International, 333 Ravenswood Ave, Menlo Park, CA 94025 USA; 3 Factor-Inwentash Faculty of Social Work, University of Toronto, 246 Bloor St W, Toronto, ON M5S 1V4 Canada; 4 Department of Management, Policy and Community Health, University of Texas Health Science Center at Houston, 1200 Pressler Street, Houston, TX 77030 USA; 5 School of Humanities and Social Science, The Chinese University of Hong Kong, 2001 Longxiang Boulevard, Longgang District, Shenzhen, Guangdong 518172 China; 6 School of Physiology, University of the Witwatersrand, 1 Jan Smuts Ave, Braamfontein, Johannesburg, 2000 South Africa

**Keywords:** Media, Parenting, Social media, Screens, Mature content, Adolescent, Epidemiology

## Abstract

**Objective:**

To assess whether specific parent media practices are associated with the consumption of R-rated (restricted) movies and mature-rated video game use in early adolescents.

**Methods:**

Data from the Adolescent Brain Cognitive Development (ABCD) Study (*N* = 10,054, 12–13 years, Year 3, 2019–2021) were analyzed. Ordinal logistic regression models were used to assess associations among media parenting practices and R-rated movies or mature-rated video game use, adjusting for potential confounders.

**Results:**

Parental allowance of bedroom screen use (adjusted odds ratio [AOR] 1.44, 95% confidence interval [CI] 1.36–1.53), family mealtime screen use (AOR 1.19, 95% CI 1.13–1.25), and parent screen use (AOR 1.11, 95% CI 1.03–1.20) were positively associated with watching R-rated movies. Parental allowance of bedroom screen use (AOR 1.44, 95% CI 1.36–1.52), family mealtime screen use (AOR 1.26, 95% CI 1.19–1.32), and parent screen use (AOR 1.11, 95% CI 1.02–1.20) were positively associated with playing mature-rated video games. Greater parental monitoring and limiting of screen time were negatively associated with watching R-rated movies (AOR 0.81, 95% CI 0.77–0.85 and AOR 0.73, 95% CI 0.68–0.79 respectively) and playing mature-rated video games (AOR 0.81, 95% CI 0.77–0.86 and AOR 0.72, 95% CI 0.67–0.77). Restricting screen time as a punishment for misbehavior was linked to a higher odds of watching R-rated movies (AOR 1.06, 95% CI 1.01–1.11) and playing mature-rated video games (AOR 1.12, 95% CI 1.07–1.17) while offering screen time to reward for good behavior was negatively associated with watching R-rated movies (AOR 0.95, 95% CI 0.90–0.99).

**Conclusions:**

Media parenting practices such as monitoring or limiting screen use are significantly associated with playing mature-rated video games and watching R-rated movies. Punitive measures, such as restricting screen time as a punishment are slightly associated with increased engagement with such content. These findings highlight the importance of intentional and thoughtful parental strategies in managing children’s media consumption effectively.

**Supplementary Information:**

The online version contains supplementary material available at 10.1186/s12887-024-05367-w.

## Introduction

R-rated (i.e., suitable for those under 17 years old when accompanied by parents or other guardians) movies and mature video games consumption in adolescents may lead to adverse outcomes in various aspects of their lives — poor mental health, worse school performance, substance use, and problematic behaviors — raising parental and public health concerns [1–3]. Early adolescence is a time in which media use often increases and establishes patterns for future habits and behaviors [4].

Developmentally, early adolescence is a period that can lead to shifting parent-adolescent relationships due to children’s increased desire for independence [5]. Given this, it is relevant to investigate how parent media practices (i.e., parenting approaches specific to technology use) influence children’s access to R-rated movies and mature-rated video games. Studies have shown that parent media practices, child-parent relationships, and home environment are critical factors in children’s screen use patterns [6, 7]. For example, parental restrictions on children’s mature-rated video game play predicted less fighting and violent behavior [9]. Parent limit setting on screens was associated with less problematic screen use [10]. One cross-sectional study found that parental restriction on mature-rated media was strongly protective for substance use outcomes such as the initiation of drinking, smoking, and illicit drug use [11].

The American Academy of Pediatrics (AAP) Parents’ Family Media Use Plan outlines several actions parents can take to reduce screen time and problematic media exposure. These parent media practices include parent modeling, bedroom screen use, mealtime screen use, parent control, parent limits, and parent monitoring [10]. Social Learning Theory, which posits that learning occurs through the observation of the consequences of others’ behaviors, may help explain potential associations found with parent modeling as adolescents may imitate and adopt behavior demonstrated by their parents [12]. Although significant associations have been found between some of the aforementioned parent media practices and increased general screen time in early adolescents, associations between all these practices and R-rated movies and mature-rated videos have not been investigated [10]. Previous literature analyzing mature content exposure looked at associations with only one specific parent media practice or used a cohort of young children or older adolescents/young adults [9, 13].

To address these gaps in the literature, our study aims to assess the specific parent media practices associated with greater consumption of R-rated movies and mature-rated video game use in a diverse national sample of early adolescents in the United States (US). We hypothesize that more parental engagement and closer monitoring of screen use are associated with a lower odds of accessing mature content through R-rated movies and mature-rated video games among early adolescents.

## Methods

### Study population

The current study utilized data from the Adolescent Brain Cognitive Development (ABCD) Study, a longitudinal cohort study tracking the health and cognitive development of 11,875 adolescents from 21 research sites across the US (baseline 2016–2018). Informed consent was obtained from caregivers, and assent was secured from each participating adolescent. The University of California, San Diego institutional review board (IRB) approved the study (160091), and local IRB approval was granted at each participating site. Further details on the structure and recruitment of participants are outlined elsewhere [14].

The baseline ABCD Study sample included 11,875 participants. As parent media parenting practice questions were only added beginning in Year 3, this study utilized data from 10,072 participants with follow-up data at Year 3 in the ABCD 5.1 release (2019–2021). From this Year 3 sample, one (1) individual without any R-rated movie consumption and mature-rated video game frequency data was excluded from the analytic sample (individuals with partial data were not excluded). Additionally, individuals who did not have propensity scores were excluded (*n* = 17), resulting in a final dataset of 10,054 adolescents included in the current analyses (see Appendix A for included (*N* = 10,054) versus excluded (due to loss-to-follow-up or missing data; *N* = 1,821) individuals from the larger baseline ABCD sample of 11,875 individuals).

## Measures

### Media parenting practices

Parents completed a 14-item self-report questionnaire to assess their typical screen time practices with their children [15]. This questionnaire used items from a previously validated measure of media parenting practices [16]. Responses were rated on a 4-point Likert-type scale from 1 (*Strongly Disagree*) to 4 (*Strongly Agree*). As with prior studies, responses to the question “I try to limit how much I use a screen-based device when I am with my child” were reverse-coded to maintain consistency in the directionality of the responses with the other question related to parental screen time modeling (Appendix B).

Responses were aggregated into six categories: (1) screen time modeling (e.g., “I try to limit how much I use a screen-based device when I am with my child”), (2) mealtime screen use (e.g., “Our family often watches a screen during meals”), (3) bedroom screen use (e.g., “My child falls asleep while using a screen-based device”), (4) use of screens to control behavior (e.g., “I offer screen time to my child as a reward for good behavior”), (5) parental monitoring of screen time (e.g., “I keep track of my child’s screen time during the week”), and (6) parental limiting of screen time (e.g., “I limit my child’s screen time during the week”) [16]. Category scores were generated by taking the average score across questions in each category. The overall parent media practices questionnaire demonstrated a McDonald’s Omega of 0.88, suggesting high internal consistency [17]. Additionally, each of the six categories of the parent media practices questionnaire was significantly correlated with total adolescent self-reported screen time. Total adolescent self-reported screen time itself was significantly positively correlated (*r* = 0.49, *p* < 0.001) with passive, objectively sensed smartphone use in the ABCD Study [18]. Correlations between items on the parent media practices questionnaire can be found in Appendix C.

### Adolescent mature-content media consumption

Adolescents self-reported their consumption of mature-content media using two questionnaire items. One item asked, “How often do you watch R-rated movies?” with potential responses being on a scale from 0 (*Never*) to 3 (*All the time*), and another asked, “How often do you play mature-rated video games (e.g., Call of Duty, Grand Theft Auto, Assassin’s Creed, etc.)?” with responses being on the same scale. Each item was treated as a separate dependent variable.

### Statistical analyses

Statistical analyses were performed using R version 4.3.3. Descriptive statistics of our data, such as means, standard deviations, and percentages, were calculated. Separate ordinal logistic regression models were created for each of the six parent media monitoring behaviors and then used to evaluate the associations between each of those behaviors and the consumption of R-rated movies and mature-rated video games. Models were fit using the *survey* package for R, version 4.4-2. Each model was adjusted for sociodemographic covariates, including age, sex assigned at birth, race/ethnicity, parental education, household income, parental marital status, and research site in accordance with prior research [10, 19, 20]. Since Year 3 data includes responses from before the COVID-19 pandemic and after the onset, an indicator variable was included as a covariate to adjust for potential history effects. Adjusted models did not demonstrate evidence of multicollinearity (all variance inflation factors < 5). Additionally, to control for potential sources of selection bias, align our sample with demographic profiles represented in the American Community Survey, and enhance the representativeness of our findings, all regression models incorporated the ABCD propensity weights [21]. Additionally, interactions with parent media practices and sex, parental education, and household income were tested with the outcomes (R-rated movies and mature video games) in adjusted models. Where there was a significant interaction by sex, parental education, or household income, stratified results are presented.

## Results

As shown in Table 1, this analysis included 10,054 eligible study participants (mean age 12.9 years, standard deviation 1.04), with 48.3% being female and 45.6% being from racial/ethnic minority groups. Unadjusted mean media parenting practice scores for our sample tended to be higher (> 2 out of 4 points) for parental screen time modeling (more likely to use screens around their children), use of screens to control behavior (more likely to treat screen time as a reward or punishment), parental monitoring of screen time (more likely to monitor child’s screen time), and limiting screen time (more likely to limit child’s screen time).

**Table 1:** Sociodemographic characteristics and media parenting practices in the Adolescent Brain Cognitive Development (ABCD) Study in Year 3 (*N* = 10,054)

Sociodemographic characteristics	Mean (SD) / %
Age (years)	12.9 (1.04)
Sex (%)	
Male	51.7%
Female	48.3%
Race/ethnicity (%)	
White	54.4%
Latino	19.9%
Black	15.6%
Asian	5.5%
Native American	3.2%
Other	1.4%
Household income (%)	
Equal to or greater than $75,000	63.9%
Less than $75,000	36.1%
Parents’ highest education (%)	
More than high school	82.2%
High school or less	17.8%
Parents’ marital status (%)	
Married or living with partner	69.3%
Unmarried or unpartnered	30.7%
Media parenting practices^1^	
Parental screen time modeling (mean score)	2.30 (0.60)
Mealtime screen use (mean score)	1.90 (0.95)
Bedroom screen use (mean score)	1.87 (0.91)
Use of screens to control behavior (mean score)	2.57 (0.88)
Parental monitoring of screen time (mean score)	2.75 (0.96)
Limiting screen time (mean score)	3.14 (0.71)
COVID pandemic	
Before COVID	15.5%
During or after COVID	84.5%

ABCD Study propensity weights were applied based on the American Community Survey from the US Census

^1^ Mean of scores for questions within each category (see Appendix B), ranging from 1 (*Strongly Disagree*) to 4 (*Strongly Agree*)

Table 2 shows the associations between media parenting practices and the frequency of playing mature-rated video games and watching R-rated movies. Greater parental screen time modeling, such as a parents’ own use of a screen-based device in their child’s presence, was significantly associated with a higher odds of children playing mature-content video games (adjusted odds ratio [AOR] 1.11; 95% confidence interval [CI] 1.02–1.20) and higher odds of watching more R-rated movies (AOR 1.11; 95% CI 1.03–1.20). Higher screen use during mealtimes was significantly associated with higher odds of reporting higher levels of mature video game use (AOR 1.26; 95% CI 1.19–1.32) and higher R-rated movie consumption (AOR 1.19; 95% CI 1.13–1.25). Additionally, higher bedroom screen use was significantly associated with higher odds of reporting mature-rated video game use (AOR 1.44; 95% CI 1.36–1.52) and more watching of R-rated movies (AOR 1.44; 1.36–1.53). Use of screens to control behavior (e.g., using screen time as a reward or punishment) was also significantly associated with higher odds of reporting mature video game use (AOR 1.08; 95% CI 1.02–1.15) but was not associated with R-rated movie consumption (AOR 1.00; 95% CI 0.95–1.06). When this control subcategory was analyzed separately (using screen time as a reward versus as a punishment, Appendix D), we found a slight difference in associations: use of screen time as a reward was associated with lower odds of reporting R-rated movie consumption. In comparison, the use of screen time as a punishment was associated with higher odds of both forms of mature media consumption. Higher parental monitoring and rules (i.e., limiting screen time) regarding adolescent screen time were associated with lower odds of adolescent engagement with mature video games and R-rated movies.

**Table 2:** Associations between media parenting practices and mature video game frequency / R-rated movie consumption in the Adolescent Brain Cognitive Development (ABCD) Study (*N* = 10,054)

Media parenting practice categories	Frequency of Mature Video Games*	*R*-rated Movies Consumption**
	*Adjusted Odds Ratio (95% Confidence Interval)*	*Adjusted Odds Ratio (95% Confidence Interval)*
Parental screen time modeling	**1.11 (1.02**,** 1.20)**	**1.11 (1.03**,** 1.20)**
Mealtime screen use	**1.26 (1.19**,** 1.32)**	**1.19 (1.13**,** 1.25)**
Bedroom screen use	**1.44 (1.36**,** 1.52)**	**1.44 (1.36**,** 1.53)**
Use of screens to control behavior	**1.08 (1.02**,** 1.15)**	1.00 (0.95, 1.06)
Parental monitoring of screen time	**0.81 (0.77**,** 0.86)**	**0.81 (0.77**,** 0.85)**
Limiting screen time	**0.72 (0.67**,** 0.77)**	**0.73 (0.68**,** 0.79)**

ABCD Study propensity weights were applied based on the American Community Survey from the US Census

* All models adjusted for age, sex assigned at birth, race/ethnicity, household income, parental education, parental marital status, study site, and an indicator of whether data was collected before or after the COVID-19 pandemic

** All models adjusted for age, sex assigned at birth, race/ethnicity, household income, parental education, parental marital status, study site, and an indicator of whether data was collected before or after the COVID-19 pandemic

Additionally, for outcomes where there were significant interactions between parent media practices and sex, parental education, or household income, we stratified results by the respective variable (Appendices E-G). Sex-stratified models showed that associations between certain media parenting practices (bedroom screen use, parental monitoring, and setting limits) and mature video games were stronger in males compared to females (Appendix E). Parental education-stratified models showed that associations between certain media parenting practices (e.g., setting limits) and mature video games and R-rated movies were stronger in households with higher than lower parental education (Appendix F). Household income-stratified models showed that associations between certain media parenting practices (e.g., bedroom screen use, setting limits) and mature video games and R-rated movies were stronger in households with higher than lower income (Appendix G).

## Discussion

In this demographically diverse sample of 10,054 early adolescents aged 12–13, parental modeling of screen use and their allowance of bedtime and mealtime screen use for their child was associated with higher mature video game use and R-rated movie consumption in adolescents. The use of screen time to control behavior, particularly taking screens away as punishment, was associated with a higher odds of mature media use. In contrast, parental monitoring and limiting adolescent screen time were generally associated with lower mature video game use and R-rated movie consumption in adolescents.

### Screen time modeling

Our study findings indicate that parental modeling of their own screen use when with their child was associated with higher mature video game use and higher R-rated movie consumption in adolescents. These findings were consistent with studies that found parental modeling to be an important target variable in interventions aiming to reduce adolescents’ prospective time spent on TV [22]. Previous literature suggests that children mirror parental behavior and learn by observing their parents’ behavior, as outlined in the social learning theory [23]. Additionally, studies have shown that children could model their parents’ screen use behaviors at home [7]. More frequent co-use of screens with children as well as greater parental screen use have both been shown to be associated with higher screen time in children [24–26]. Additionally, parents who consume media more frequently are possibly more open to children’s access to media and may impose fewer restrictions on their adolescents [24].

### Mealtime & bedroom screen use

Our analysis suggests that family screen use during mealtime and access to screens in the child’s bedroom are significantly associated with higher odds of reporting greater mature video game use and R-rated movie consumption. Bedroom screen use emerged as the strongest predictor of mature media consumption in our analysis. Possibly, more access to screens, especially if in the privacy of the bedroom, allows unchecked exposure to mature content. Our findings support research that shows the adverse health effects of using screens during meals and at bedtime. Specifically, screen use during meals has been linked to emotional, behavioral, and dietary problems, and screen use at bedtime has been linked to obesity, shorter sleep duration, and poor sleep quality among children [27–29]. Additionally, studies investigating parental practices regarding bedtime screen access for their child showed that adolescents with greater exposure to R-rated movies and mature video games were significantly more likely to have a television in their bedroom and report that their parents allowed them to watch R-rated movies [8] These findings reinforce guidance from the American Academy of Pediatrics that encourages parents to make mealtimes and bedtimes screen-free. Further work is warranted to understand the relationship between family screen use during mealtime and bedtime screen access and mature video games and R-rated movie consumption among adolescents in more detail.

### Screens to control behavior

Prior research has found that the use of screens to control behavior was associated with greater adolescent screen time [10, 15, 30, 31], but there has been little to no research on how the use of screen time as a reward or punishment to control behavior relates to adolescent consumption of mature media. We analyzed this association by grouping both questions under the “use of screens to control behavior” category together and then looking at reward and punishment separately. When aggregated, we found that higher use of screen time to control behavior, in general, was associated with greater odds of adolescents using screen time for mature video games but not R-rated movies. When the two questions in this category were analyzed separately, the use of screen time as a reward was found to be associated with lower odds of R-rated movie consumption, which is consistent with prominent behavioral theories and research linking positive reinforcement to increases in desirable behavior [32]. However, using external rewards like more screen time to encourage positive behavior may not be associated with the desired outcome if the external reward is perceived to be more valuable than the desired behavior [32]. On the other hand, findings regarding not limiting screen time as a form of punishment are consistent with the concept of reactance theory relating to adolescents’ response to being punished in this manner [33]; alternatively, our observations could also reflect parents’ removal of screen time in response to discovering their adolescents using screen time for mature content. Regardless, it is worth considering the effect of parenting style on child behavior in the context of screen use for mature media, as previous studies have associated autonomy-supporting parenting styles with an increase in desirable adolescent screen use behaviors and controlling parenting styles with an increase in undesirable adolescent screen use behaviors [7, 34].

### Monitoring screen time & limiting screen time

Parental monitoring of adolescent screen time and proactively limiting screen use were significantly associated with lower odds of adolescents consuming media with mature content. This finding aligns with previous research, which identified an association between parental monitoring behavior of screen use and the time spent playing video games [34, 35] and media violence exposure [36]. Possibly, screen-related monitoring behavior may curb adolescent consumption of mature-related media [37]. It is also noteworthy that parental monitoring of screen time was more strongly associated with lower mature video game use and R-rated movies in males compared to females. Limiting screen time was more strongly associated with consuming media with mature content in households with higher parent education and higher income. Future research may develop more effective guidance for parents from lower socioeconomic backgrounds.

### Limitations and strengths

Limitations of this study should be noted. Given that this study is cross-sectional, causality cannot be determined. Additionally, longitudinal data about parent media practices are not yet available for the ABCD Study, therefore, we were not able to conduct analyses that require longitudinal data, such as prospective associations with outcomes or mediation analyses. Due to the self-reported nature of the data, there is a possibility of reporting and recall bias [38]. Further, there is a possibility of selection bias due to the significant differences between included and excluded individuals in our study. Specifically, individuals of lower socioeconomic status and who identify as Latino or Black are underrepresented in our study population. Additionally, although we did adjust for potential confounders, such as race, sex, and income, it is possible that there are residual confounders that were not accounted for, such as adolescent behaviors, parenting styles, and video game addiction [38]. As literature shows that screen time among adolescents during the COVID-19 pandemic increased by 52% [39], it is also important to consider that the data used in this study (Year 3 data from ABCD) overlaps with the years of the COVID-19 pandemic. We adjusted for this variable in the statistical analysis; however, the shifting of family dynamics and the social distancing restrictions leading to decreased in-person interactions and increased screen time should be noted when interpreting the results. Additionally, it should be noted that all odds ratios (ORs), though significant, are relatively small. Finally, mature content ratings are not limited to video games and movies and are applied to social media as well. The ABCD Study does not contain information on the consumption of mature-rated social media, which could fail to capture all mature content consumption among adolescents. Similarly, the ABCD Study does not contain information on whether adolescents consume media in the presence of others (e.g., family or friends), which could mediate the relationship between parent media practices and mature media consumption.

Notable strengths of this study include our large, national, diverse sample of early adolescents in the United States and measures of parent media practices and R-rated movie and mature-rated video game exposure based on both parent and adolescent reports. To our knowledge, our study is the first to assess multiple parent media practices in relation to R-rated movies and mature video game consumption in a sample of demographically diverse early adolescents in the US.

### Future research directions

Future research can use longitudinal data to explore to what extent parent media practices may explain the relationships between exposure to mature-content movies and video games and the potential negative outcomes shown in other studies (e.g., school performance), which have suffered in children whose parents allowed them to watch R-rated movies [1]. For example, previous literature has found watching R-rated movies predicted a greater odds of smoking initiation, which was further enhanced by private access to a television during early adolescence, such as having a bedroom television [2]. Playing mature and violent-content video games was associated with higher depressive symptoms, problematic internet use, decreased sleep, increased time spent playing games, and a higher frequency of checking social media as compared to the adolescent students who did not play these video games [3]. Studies have also found a positive association between adolescent exposure to violent video games and aggressive behaviors later in life [40–42]. Given these findings and the results of our stratified and interaction term analyses, more research is needed to explore the relationship between key sociodemographic covariates and mature video games and R-rated movie consumption as well as the negative outcomes that have been shown to have associations.

## Conclusion

The current study has several implications given the potential risks associated with R-rated movies and mature video game consumption in adolescents combined with the increasing number of early adolescents consuming media. Our findings suggest that parental monitoring and limiting of adolescent screen time are significantly associated with lower mature video game use and R-rated movie consumption in adolescents. In contrast, parental modeling of their own screen use and allowance of mealtime and bedtime screen use were significantly associated with higher mature video game use and R-rated movie consumption in adolescents. These findings can further inform guidelines such as those outlined by the American Academy of Pediatrics Family Media Use Plans [4]. Findings show that clear and consistent rules that are agreed upon by parents and children can make the implementation of guidelines such as the Family Media Use plan more effective and successful in the long term [35, 43, 44]. Future research may explore more objective measures of negative behaviors associated with R-rated movie and mature video game consumption in early adolescents and media parenting practices specific to mature content exposure to further inform clinical guidelines and public health policies for early adolescents.

## Electronic supplementary material

Below is the link to the electronic supplementary material.

Additional File 1: Appendix A, Appendix B, Appendix C, Appendix D, Appendix E, Appendix F, Appendix G. Description of data: Appendix (A) Comparison of sociodemographic characteristics between included and excluded Adolescent Brain Cognitive Development (ABCD) Study participants; Appendix (B) Description of parenting media practice items in the Adolescent Brain Cognitive Development (ABCD) Study; Appendix (C) Correlation table between parent media practices in the Adolescent Brain Cognitive Development (ABCD) Study; Appendix (D) Associations between media parenting practices that reward or punish their children and mature video game frequency / R-rated movie consumption in the Adolescent Brain Cognitive Development (ABCD) Study (*N* = 10,054). Appendix (E) Associations between media parenting practices and mature video game frequency / R-rated movie consumption in the Adolescent Brain Cognitive Development (ABCD) Study, stratified by sex (*N* = 10,054). Appendix (F) Associations between media parenting practices and mature video game frequency / R-rated movie consumption in the Adolescent Brain Cognitive Development (ABCD) Study, stratified by parental education (*N* = 10,054). Appendix (G) Associations between media parenting practices and mature video game frequency / R-rated movie consumption in the Adolescent Brain Cognitive Development (ABCD) Study, stratified by household income (*N* = 10,054)

## Data Availability

Data used in the preparation of this article were obtained from the ABCD Study (https://abcdstudy.org), held in the NIMH Data Archive (NDA). R Code is available at https://github.com/jasonmnagata/ABCD-Study/blob/main/parentmediamature.

## Abbreviations

AAP: American Academy of Pediatrics
ABCD: Adolescent Brain Cognitive Development Study
