# Supplementary material for: Associations between media parenting practices and early adolescent consumption of R-rated movies and mature-rated video games
Source: BMC Pediatr. 2025 Feb 4;25:90. doi: 10.1186/s12887-024-05367-w (PMC11792743; doi:10.1186/s12887-024-05367-w)
Supplement: Supplementary file 1 — Additional File 1: Appendix A, Appendix B, Appendix C, Appendix D, Appendix E, Appendix F, Appendix G. Description of data: Appendix (A) Comparison of sociodemographic characteristics between included and excluded Adolescent Brain Cognitive Development (ABCD) Study participants; Appendix (B) Description of parenting media practice items in the Adolescent Brain Cognitive Development (ABCD) Study; Appendix (C) Correlation table between parent media practices in the Adolescent Brain Cognitive Development (ABCD) Study; Appendix (D) Associations between media parenting practices that reward or punish their children and mature video game frequency / R-rated movie consumption in the Adolescent Brain Cognitive Development (ABCD) Study (N = 10,054). Appendix (E) Associations between media parenting practices and mature video game frequency / R-rated movie consumption in the Adolescent Brain Cognitive Development (ABCD) Study, stratified by sex (N = 10,054). Appendix (F) Associations between media parenting practices and mature video game frequency / R-rated movie consumption in the Adolescent Brain Cognitive Development (ABCD) Study, stratified by parental education (N = 10,054). Appendix (G) Associations between media parenting practices and mature video game frequency / R-rated movie consumption in the Adolescent Brain Cognitive Development (ABCD) Study, stratified by household income (N = 10,054) [file 12887_2024_5367_MOESM1_ESM.docx]

**Online Supplement**

Appendix A. Comparison of sociodemographic characteristics between included and excluded Adolescent Brain Cognitive Development (ABCD) study participants.

| Sociodemographic characteristics | Excluded*^1^*  (N = 1,821) | Included*^1^*  (N = 10,054) | p-value*^2^* |
| --- | --- | --- | --- |
| Age (years) | 12.03 (3.61) | 12.90 (1.04) | 0.8 |
| Sex (%) |  |  | 0.026 |
| Male | 48.5% | 51.7% |  |
| Female | 51.5% | 48.3% |  |
| Race/ethnicity (%) |  |  | <0.001 |
| White | 41.7% | 54.4% |  |
| Latino | 21.3% | 19.9% |  |
| Black | 26.8% | 15.6% |  |
| Asian | 5.4% | 5.5% |  |
| Native American | 3.2% | 3.2% |  |
| Other | 1.5% | 1.4% |  |
| Household income (%) |  |  | <0.001 |
| Equal to or greater than $75,000 | 44.5% | 63.9% |  |
| Less than $75,000 | 55.5% | 36.1% |  |
| Parents’ highest education (%) |  |  | <0.001 |
| More than high school | 67.0% | 82.2% |  |
| High school or less | 33.0% | 17.8% |  |
| Parents’ marital status (%) |  |  | <0.001 |
| Married or living with partner | 56.1% | 69.3% |  |
| Unmarried or unpartnered | 43.9% | 30.7% |  |
| COVID pandemic |  |  | <0.001 |
| Before COVID | 1.5% | 15.5% |  |
| During or after COVID | 98.5% | 84.5% |  |
| ABCD Study propensity weights were applied based on the American Community Survey from the US Census. | | | |
| *^1^* Mean (SD); % | | | |
| *^2^* Design-based Kruskal Wallis test; Pearson’s chi-square test: Rao & Scott adjustment | | | |

| ​Appendix B. Description of parenting media practice items in the Adolescent Brain Cognitive Development (ABCD) Study. |
| --- |
| *Description of media parenting practices items* |
| **Parental screen time modeling** |
| When I am with my child, I use a screen-based device |
| I try to limit how much I use a screen-based device when I am with my child^1^ |
| **Mealtime screen use** |
| Our family often watches a screen during meals |
| Family members are allowed to use screen-based devices during meals |
| **Bedroom screen use** |
| My child falls asleep while using a screen-based device |
| A screen-based device is usually playing in the room when my child falls asleep |
| My child has access to a mobile screen-based device in bed |
| **Use of screens to control behavior** |
| I offer screen time to my child as a reward for good behavior |
| I take away screen time from my child as a punishment for bad behavior |
| **Parental monitoring of screen time** |
| I keep track of my child's screen time during the week |
| I keep track of my child's screen time during the weekend |
| **Limiting screen time** |
| I limit my child's screen time during the week |
| I limit my child's screen time during the weekend |
| I encourage my child to do activities other than screen time |

Questions originate from the ABCD study’s parental questionnaire on screen time use. Responses were rated on a 4-point Likert-type scale from 1 (*Strongly Disagree*) to 4 (*Strongly Agree*).

^1^The score for this question was reverse coded for consistency in the directionality of the parental screen time modeling questions in the regression model.

Appendix C. Correlation table between parent media practices in the Adolescent Brain Cognitive Development (ABCD) study (N = 10,054).

|  | Parental screen time modeling | Mealtime screen use | Bedroom screen use | Use of screens to control behavior | Parental monitoring of screen time | Limiting screen time |
| --- | --- | --- | --- | --- | --- | --- |
| Parental screen time modeling | - | 0.16 | 0.10 | 0.00 | -0.20 | -0.21 |
| Mealtime screen use | 0.16 | - | 0.38 | 0.09 | -0.15 | -0.19 |
| Bedroom screen use | 0.10 | 0.38 | - | 0.08 | -0.22 | -0.28 |
| Use of screens to control behavior | 0.00 | 0.09 | 0.08 | - | 0.27 | 0.29 |
| Parental monitoring of screen time | -0.20 | -0.15 | -0.22 | 0.27 | - | 0.70 |
| Limiting screen time | -0.21 | -0.19 | -0.28 | 0.29 | 0.70 | - |

Appendix D. Associations between media parenting practices that reward or punish their children and mature video game frequency / R-rated movie consumption in the Adolescent Brain Cognitive Development (ABCD) study (N = 10,054).

| Media parenting practice question | Frequency of Mature Video Games* | R-rated Movies Consumption** |
| --- | --- | --- |
|  | Adjusted Odds Ratio  (95% CI) | Adjusted Odds Ratio  (95% CI) |
| I offer screen time to my child as a reward for good behavior. | 1.00 (0.96, 1.05) | **0.95 (0.91, 0.99)** |
| I take away screen time from my child as a punishment for bad behavior. | **1.12 (1.07, 1.18)** | **1.06 (1.01, 1.11)** |
| ABCD Study propensity weights were applied based on the American Community Survey from the US Census.  * All models adjusted for age, sex assigned at birth, race/ethnicity, household income, parental education, parental marital status, study site, and an indicator of whether data was collected before or after the COVID-19 pandemic.  ** All models adjusted for age, sex assigned at birth, race/ethnicity, household income, parental education, parental marital status, study site, and an indicator of whether data was collected before or after the COVID-19 pandemic. | | |

| Appendix E. Associations between media parenting practices and mature video game frequency / R-rated movie consumption in the Adolescent Brain Cognitive Development (ABCD) Study, stratified by sex (N = 10,054). | | | | | | | | | | |
| --- | --- | --- | --- | --- | --- | --- | --- | --- | --- | --- |
|  | Mature Video Games* | | | | | R-rated Movies** | | | | |
|  | Female (N = 4772) | | | Male (N = 5280) | | Female (N = 4772) | | | Male (N = 5280) | |
|  | *Adjusted Odds Ratio*  *(95% CI)* | *p-value* | *p-value for interaction* | *Adjusted Odds Ratio*  *(95% CI)* | *p-value* | *Adjusted Odds Ratio*  *(95% CI)* | *p-value* | *p-value for interaction* | *Adjusted Odds Ratio*  *(95% CI)* | *p-value* |
| Modeling | --- | --- | 0.20 | --- | --- | --- | --- | 0.53 | --- | --- |
| Mealtime | **----** | **---** | 0.55 | **---** | **---** | **---** | **---** | 0.14 | **---** | **---** |
| Bedroom | **1.34 (1.23, 1.46)** | **<0.001** | **0.006** | **1.48 (1.38, 1.59)** | **<0.001** | **---** | **---** | 0.35 | **---** | **---** |
| Control | --- | --- | 0.43 | --- | --- | --- | --- | 0.11 | --- | --- |
| Monitoring | **0.87 (0.80, 0.94)** | **<0.001** | **0.003** | **0.78 (0.73, 0.84)** | **0.02** | **0.86 (0.80, 0.92)** | **<0.001** | **0.02** | **0.77 (0.72, 0.83)** | **<0.001** |
| Limits | **0.78 (0.71, 0.87)** | **<0.001** | **0.004** | **0.68 (0.62, 0.75)** | **<0.001** | **---** | **---** | 0.10 | **---** | **---** |
| ABCD Study propensity weights were applied based on the American Community Survey from the US Census.  * All models adjusted for age, race/ethnicity, household income, parental education, parental marital status, study site, and an indicator of whether data was collected before or after the COVID-19 pandemic.  ** All models adjusted for age, race/ethnicity, household income, parental education, parental marital status, study site, and an indicator of whether data was collected before or after the COVID-19 pandemic. | | | | | | | | | | |

| Appendix F. Associations between media parenting practices and mature video game frequency / R-rated movie consumption in the Adolescent Brain Cognitive Development (ABCD) Study, stratified by parental education (N = 10,054). | | | | | | | | | | |
| --- | --- | --- | --- | --- | --- | --- | --- | --- | --- | --- |
|  | Mature Video Games* | | | | | R-rated Movies** | | | | |
|  | High school or less  (N = 1508) | | | More than high school  (N = 8534) | | High school or less  (N = 1508) | | | More than high school  (N = 8534) | |
|  | *Adjusted Odds Ratio*  *(95% CI)* | *p-value* | *p-value for interaction* | *Adjusted Odds Ratio*  *(95% CI)* | *p-value* | *Adjusted Odds Ratio*  *(95% CI)* | *p-value* | *p-value for interaction* | *Adjusted Odds Ratio*  *(95% CI)* | *p-value* |
| Modeling | --- | --- | 0.89 | **---** | **---** | **---** | **---** | 0.43 | **---** | **---** |
| Mealtime | 1.03 (0.92, 1.16) | 0.59 | **0.002** | **1.30 (1.23, 1.38)** | **<0.001** | **---** | **---** | 0.91 | **---** | **---** |
| Bedroom | **---** | **---** | 0.05 | **---** | **---** | **---** | **---** | 0.09 | **---** | **---** |
| Control | --- | --- | 0.99 | **---** | **---** | --- | --- | 0.90 | --- | --- |
| Monitoring | --- | --- | 0.29 | **---** | **---** | 0.96 (0.85, 1.09) | 0.54 | **0.01** | **0.78 (0.74, 0.83)** | **<0.001** |
| Limits | 0.90 (0.78, 1.04) | 0.14 | **0.003** | **0.67 (0.62, 0.73)** | **<0.001** | 0.94 (0.80, 1.10) | 0.43 | **<0.001** | **0.68 (0.63, 0.74)** | **<0.001** |
| ABCD Study propensity weights were applied based on the American Community Survey from the US Census.  * All models adjusted for age, sex assigned at birth, race/ethnicity, household income, parental marital status, study site, and an indicator of whether data was collected before or after the COVID-19 pandemic.  ** All models adjusted for age, sex assigned at birth, race/ethnicity, household income, parental marital status, study site, and an indicator of whether data was collected before or after the COVID-19 pandemic. | | | | | | | | | | |

| Appendix G. Associations between media parenting practices and mature video game frequency / R-rated movie consumption in the Adolescent Brain Cognitive Development (ABCD) Study, stratified by household income (N = 10,054). | | | | | | | | | | |
| --- | --- | --- | --- | --- | --- | --- | --- | --- | --- | --- |
|  | Mature Video Games* | | | | | R-rated Movies** | | | | |
|  | Less than $75,000 (N = 2496) | | | Equal to or greater than $75,000 (N = 6797) | | Less than $75,000 (N = 2496) | | | Equal to or greater than $75,000 (N = 6797) | |
|  | *Adjusted Odds Ratio*  *(95% CI)* | *p-value* | *p-value for interaction* | *Adjusted Odds Ratio*  *(95% CI)* | *p-value* | *Adjusted Odds Ratio*  *(95% CI)* | *p-value* | *p-value for interaction* | *Adjusted Odds Ratio*  *(95% CI)* | *p-value* |
| Modeling | --- | --- | 0.39 | **---** | **---** | --- | --- | 0.40 | **---** | **---** |
| Mealtime | **1.15 (1.06, 1.26)** | **0.001** | **0.004** | **1.32 (1.24, 1.41)** | **<0.001** | **---** | **---** | 0.84 | **---** | **---** |
| Bedroom | **1.33 (1.22, 1.46)** | **<0.001** | **0.007** | **1.50 (1.40, 1.61** | **<0.001** | **1.30 (1.18, 1.42)** | **<0.001** | **0.005** | **1.54 (1.44, 1.65)** | **<0.001** |
| Control | --- | --- | 0.28 | **---** | **---** | --- | --- | 0.68 | --- | --- |
| Monitoring | **---** | **---** | 0.07 | **---** | **---** | **---** | **---** | 0.32 | **---** | **---** |
| Limits | **0.81 (0.73, 0.91)** | **<0.001** | **0.004** | **0.66 (0.61, 0.72)** | **<0.001** | **0.83 (0.74, 0.93)** | **0.002** | **0.02** | **0.68 (0.62, 0.74)** | **<0.001** |
| ABCD Study propensity weights were applied based on the American Community Survey from the US Census.  * All models adjusted for age, sex assigned at birth, race/ethnicity, parental education, parental marital status, study site, and an indicator of whether data was collected before or after the COVID-19 pandemic.  ** All models adjusted for age, sex assigned at birth, race/ethnicity, parental education, parental marital status, study site, and an indicator of whether data was collected before or after the COVID-19 pandemic. | | | | | | | | | | |
